# MCT Expression and Lactate Influx/Efflux in Tanycytes Involved in Glia-Neuron Metabolic Interaction

**DOI:** 10.1371/journal.pone.0016411

**Published:** 2011-01-28

**Authors:** Christian Cortés-Campos, Roberto Elizondo, Paula Llanos, Romina María Uranga, Francisco Nualart, María Angeles García

**Affiliations:** 1 Laboratorio de Biología Celular, Departamento de Biología Celular, Universidad de Concepción, Concepción, Chile; 2 Laboratorio de Neurobiología y Células Madre, Departamento de Biología Celular, Universidad de Concepción, Concepción, Chile; 3 Instituto de Investigaciones Bioquímicas de Bahía Blanca, Universidad Nacional del Sur y Consejo Nacional de Investigaciones Científicas y Técnicas, Bahía Blanca, Argentina; Centre National de la Recherche Scientifique, University of Bordeaux, France

## Abstract

Metabolic interaction via lactate between glial cells and neurons has been proposed as one of the mechanisms involved in hypothalamic glucosensing. We have postulated that hypothalamic glial cells, also known as tanycytes, produce lactate by glycolytic metabolism of glucose. Transfer of lactate to neighboring neurons stimulates ATP synthesis and thus contributes to their activation. Because destruction of third ventricle (III-V) tanycytes is sufficient to alter blood glucose levels and food intake in rats, it is hypothesized that tanycytes are involved in the hypothalamic glucose sensing mechanism. Here, we demonstrate the presence and function of monocarboxylate transporters (MCTs) in tanycytes. Specifically, MCT1 and MCT4 expression as well as their distribution were analyzed in Sprague Dawley rat brain, and we demonstrate that both transporters are expressed in tanycytes. Using primary tanycyte cultures, kinetic analyses and sensitivity to inhibitors were undertaken to confirm that MCT1 and MCT4 were functional for lactate influx. Additionally, physiological concentrations of glucose induced lactate efflux in cultured tanycytes, which was inhibited by classical MCT inhibitors. Because the expression of both MCT1 and MCT4 has been linked to lactate efflux, we propose that tanycytes participate in glucose sensing based on a metabolic interaction with neurons of the arcuate nucleus, which are stimulated by lactate released from MCT1 and MCT4-expressing tanycytes.

## Introduction

The ventromedial hypothalamus (VMH) is involved in the regulation of satiety and feeding behavior through its capacity to detect changes in glucose concentrations [1]. The VMH, formed by the arcuate nucleus (AN) and the ventromedial nucleus (VMN), contains both glucose-excited (GE) neurons, which increase their firing rate with increasing glucose concentrations, and glucose-inhibited (GI) neurons, which respond to increases in glucose concentration by decreasing their electrical activity [2], [3]. Current literature describes mechanisms by which GE neurons detect changes in extracellular glucose. The most studied of these mechanisms is similar to that described in pancreatic β-cells and involves glucose uptake by neuronal metabolism through glucokinase and ATP production [1], [4], [5], [6]. Recently, a non-metabolic pathway that involves the participation of sodium-dependent glucose co-transporters (SGLT) has been described [7], [8]. Furthermore, an alternative pathway that involves a metabolic interaction between AN neurons and surrounding glia via lactate has also been proposed. Different *in vitro* studies have shown that lactate can influence the behavior of GE neurons from the VMH [4], [6], suggesting that this monocarboxylate is required for glucose sensing in the brain. In this context, it has been proposed that glycolytic metabolism of glucose to lactate by hypothalamic glial cells and the subsequent release to neighboring neurons using monocarboxylate transporters (MCTs) may lead to enhanced ATP synthesis, closure of K_ATP_ channels, and neuronal depolarization [9].

MCTs are a family of transporters which mediate facilitated diffusion of lactate and several other metabolically important monocarboxylates, such as pyruvate and ketone bodies [10], [11], [12]. To date, fourteen isoforms of MCTs have been identified [10], [11], [12], [13], [14]. Protein and mRNA expression studies have shown elevated MCT1 and MCT2 expression levels in the central nervous system. MCT1 has a widespread distribution; its expression has been detected both in lactate-producing and lactate-consuming tissues (e.g., erythrocytes and heart, respectively) [15]. MCT1 displays a Km of 7.7 mM for lactate influx [16]. In the brain, MCT1 has been localized in astrocytes, blood vessels, and ependymal cells [12], [17], [18], [19], [20]. MCT2 expression is mainly restricted to neurons in the cortex [20], hippocampus, and cerebellum [21], [22], [23]; it has a Km of 0.8 mM for lactate influx [10]. MCT4 has been observed in lactate-producing tissues (e.g., skeletal muscle and astrocytes) [24], [25] and displays a Km of 34 mM for the efflux of lactate [15]. Recently, MCT4 has been localized to the paraventricular nucleus, specifically in astrocytes and ciliated ependymal cells [12].

Neurons from the VMH are in close contact with highly elongated ependymal cells known as tanycytes [26], [27], which are the main glial cell present in the basal hypothalamus[28], [29], [30]. Tanycytes are classified into four different types, α1, α2, β1, and β2, according to their histological properties [30], [31], [32]. α2 and β1 tanycytes are localized in the lower lateral wall of the III-V, and they have extended cell processes that contact the neurons in the AN and VMN as well as the blood vessels in the hypothalamus and lateral median eminence (ME). We have demonstrated that these cells express proteins involved in the β-pancreatic glucose sensing mechanism. For example, the glucose transporter 2 (GLUT2) has been observed in the apical membrane of tanycytes, thus contacting the cerebrospinal fluid (CSF) [29]. Furthermore, tanycytes express glucokinase (GK) [30]. Therefore, periventricular hypothalamic tanycytes may be involved in detecting glucose concentration in the CSF of the ventricular system and generating lactate as an intercellular messenger, informing the neurons of glucose levels and regulating glucosensing activities. To test this hypothesis, we evaluated MCT1 and MCT4 expression and function in hypothalamic cells. MCT1 and MCT4 were found to be mainly expressed in tanycytes and involved in lactate influx and efflux. Taken together, these data suggest that hypothalamic tanycytes could be responsible for hypothalamic glucosensing.

## Results

### Differential distribution of MCT1 and MCT4 in the hypothalamus

**Figure 1:**
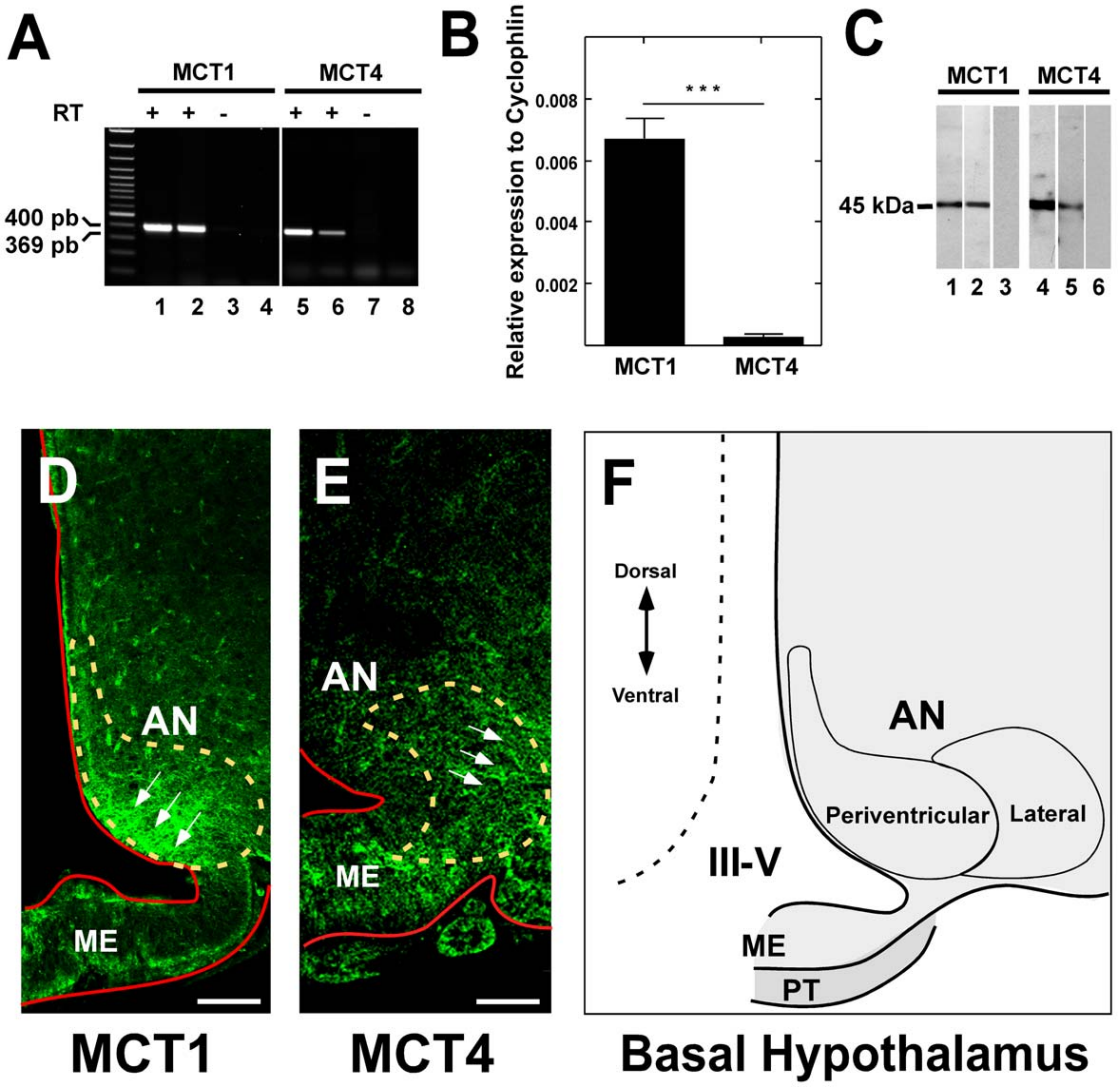
MCT1 and MCT4 expression and localization in adult rat hypothalamus. **A**, RT-PCR analysis of MCT1 and MCT4. MCT1 RT-PCR products obtained using total RNA isolated from: Lane 1, kidney cortex; 2, hypothalamus; 3 RT(-) of hypothalamus; 4, water in the PCR reaction. MCT4 RT-PCR products obtained using total RNA isolated from: Lane 5, skeletal muscle; 6, hypothalamus; 7 RT(-) of hypothalamus; 8, water in the PCR reaction. **B**, Quantitative RT-PCR analysis of the MCT1 and MCT4 mRNA levels in samples isolated from rat hypothalamus. **p<0.001. Data represent the means ± SD of the ratio of MCT mRNA to cyclophilin mRNA from three independent experiments. **C**, Western blot analysis of MCT1 and MCT4. Lanes 1–3, MCT1; 4–6, MCT4. Total protein extracts were prepared from renal cortex (lane 1), hypothalamus (lanes 2 and 5), negative control (lanes 3 and 6), and skeletal muscle (lane 4). Negative controls were performed in hypothalamus with primary antibodies preabsorbed with inductor peptides (lanes 4 and 6). **D**, Immunohistochemistry of MCT1 and confocal microscopy analysis. MCT1 is observed in the arcuate nucleus periventricular region (arrows). **E**, Immunohistochemistry of MCT4 and confocal microscopy analysis. MCT4 is localized in the arcuate nucleus lateral area (arrows). **F**, Schematic representation of basal hypothalamus. AN, arcuate nucleus; III-V, third ventricle; ME, median eminence; PT, *pars tuberalis*. Scale bar D–E, 200 µm.

MCT expression in rat hypothalamus was initially analyzed using RT-PCR with primers specific for MCT1 and MCT4 mRNAs. The conditions were optimized using RNA from the rat kidney cortex for MCT1 and skeletal muscle for MCT4. The amplified cDNA bands were 400 and 369 bp, which are the expected sizes for MCT1 and MCT4, respectively (Fig. 1A, lanes 2 and 6). No amplification product was observed in samples without reverse transcriptase, indicating the absence of DNA contamination. Quantitative RT-PCR analysis showed that MCT1 expression in hypothalamic cells is 20 times higher than MCT4 (Fig. 1B). MCT1 expression was also demonstrated using Western blot analysis of proteins isolated from rat kidney (positive control tissue) and hypothalamus (Fig. 1C, lanes 1–2). Similarly, MCT4 was evaluated using protein extracts from rat skeletal muscle (positive control tissue) and hypothalamus (Fig. 1C, lanes 4–5). Analysis of hypothalamus protein extract using anti-MCT preabsorbed with inductor peptides showed the absence of bands (Fig. 1C, lanes 3 and 6).

MCT distribution was further assessed in frontal hypothalamic sections by immunohistochemistry. The high specificity of the antibodies used in this study is shown in Figure S1; anti-MCT1 reactivity was determined in the kidney (Fig. S1A), and anti-MCT4 reactivity was evaluated in skeletal muscle (Fig. S1H). In the brain, MCT1 was primarily detected in endothelial cells, astrocytes from several regions, and in the marginal zone (Fig. S1B–G). MCT4 was detected in astrocytes from the hippocampus, *corpus callosum*, cortex, and AN (Fig. S1I–N). In the hypothalamus, MCT1 was mainly detected in the AN periventricular region (Fig. 1D and F). However, MCT4 was concentrated in the AN lateral area (Fig. 1E–F). Thus, MCT1 and MCT4 are differentially localized within the hypothalamus.

### MCT1 localization in tanycytes

**Figure 2:**
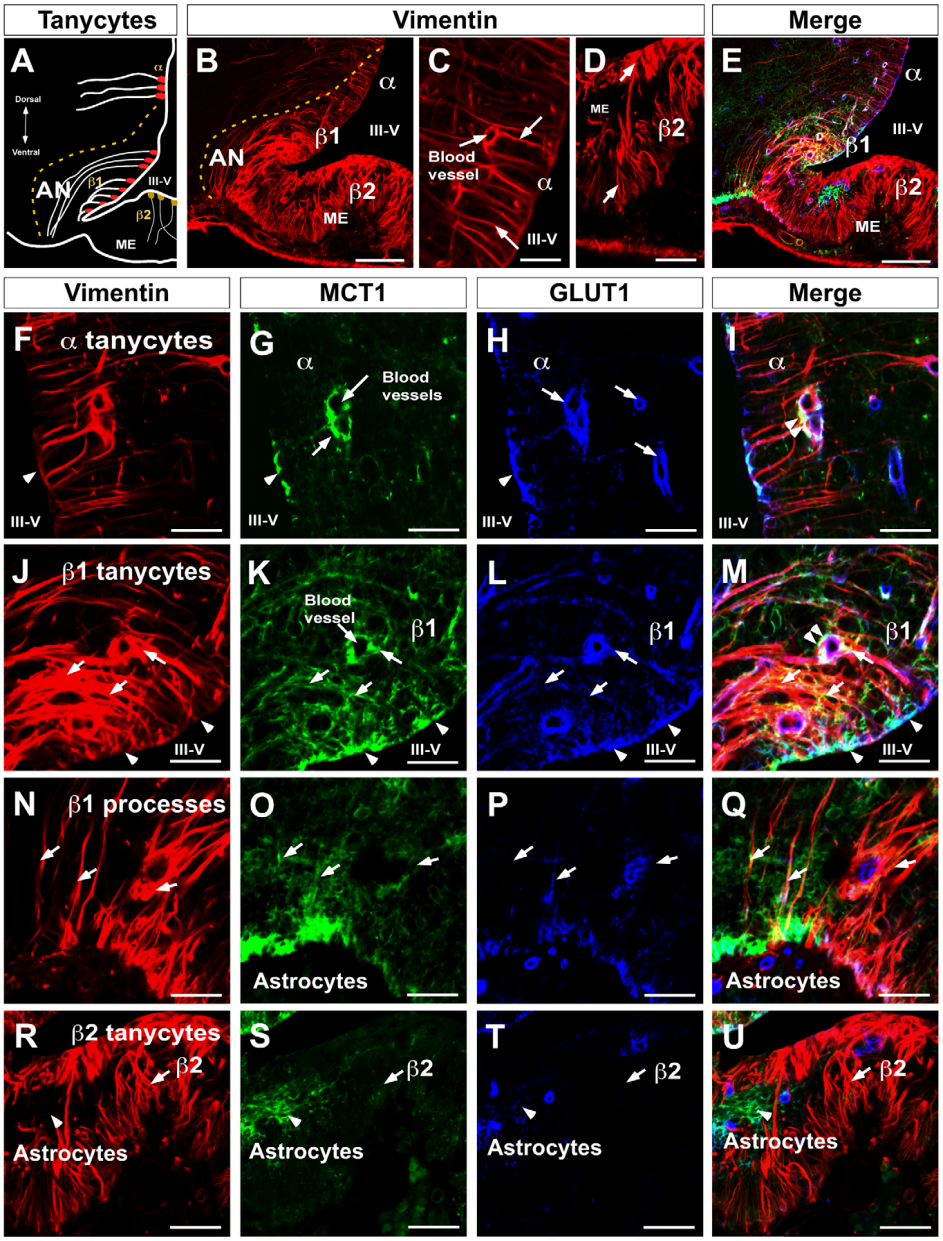
MCT1 and GLUT1 codistribution in β1-tanycytes. **A**, Schematic representation of hypothalamic area showed in B and E. **B**, rat frontal brain section using anti-vimentin antibodies. **C**, High magnification of α1-tanycytes using anti-vimentin antibodies. **D**, High magnification of β2-tanycytes using anti-vimentin antibodies. **E**, rat frontal brain section using anti-vimentin (red), anti-GLUT1 (blue), and anti-MCT1 (green) antibodies. **F–I**, α1-tanycyte area, MCT1 and GLUT1 were observed in the ventricular cellular membranes and end-feet processes contacting blood vessels. **J–M**, β1-tanycyte area, MCT1 was expressed in the proximal area of the cells colocalizing with GLUT1 and vimentin (head arrows). β1-tanycyte processes contacting periventricular AN neurons were strongly positive for MCT1 (arrows); colocalization with GLUT1 was also observed in blood vessels (arrows). **N–Q**, β1-tanycyte processes contacting the external region of the brain showed lower reaction for MCT1 and GLUT1. **R–U**, β2-tanycytes and processes in the median eminence showed negative immunoreaction for MCT1 and GLUT1. AN, arcuate nucleus, III-V: third ventricle, ME: median eminence. Scale bar: B and E, 150 µm; C–D, and F–U, 50 µm.

α and β-tanycytes were identified with an anti-vimentin antibody (Fig. 2A, B–D). α-tanycytes possessed long fine processes contacting blood vessels and VMN neurons (Fig. 2B–C, arrows). β1 and β2-tanycytes presented stronger vimentin immunoreaction and branched processes which contact both AN neurons (β1-tanycytes) or the ME (β2-tanycytes) (Fig. 2B and D, arrows). Multilabeling immunohistochemistry revealed MCT1 (green), GLUT1 (blue), and vimentin (red) colocalization (Fig. 2E). High magnification images were used to define the exact localization of the transporters in these cells. Polarized immunoreactions for MCT1 was detected in α-tanycytes (Fig. 2F–G, arrows) specifically in the ventricular cellular membranes (Fig. 2G, arrow head) and on end-feet processes contacting the endothelial cells of the blood vessels (Fig. 2G, arrow), which was similar to the pattern observed with anti-GLUT1 (Fig. 2H). However, the highest staining was found in the endothelial cells that form the blood-brain barrier (Fig. 2G, arrows). High MCT1, GLUT1, and vimentin (white colour) colocalization was detected in tanycyte end-feet processes contacting blood vessels (Fig. 2I, double arrow heads).

The strongest staining for MCT1 was concentrated in β1-tanycytes (Fig. 2J–K, arrows), particularly in the apical cellular membranes (Fig. 2K, arrow heads) and in the processes that contact AN neurons, blood vessels, and the external region of the brain (Fig. 2K and O, arrows). Colocalization of MCT1 with GLUT1 was detected in the ventricular area (Fig. 2L and M, arrow heads) and in several cell processes (Fig. 2L–M and P–Q, arrows). The Rr value estimated for MCT1/GLUT1 colocalization was 0.62±0.02 in the periventricular AN, which was statistically significant compared to the value in the lateral AN (control area) 0.2±0.02 (*p*<0.01). Intense MCT1, GLUT1, and vimentin colocalization (white) was observed in the apical tanycyte membranes and end-feet processes contacting blood vessels (Fig. 2M, double arrow heads). The Rr value estimated for MCT1/vimentin colocalization was 0.4±0.02 in the periventricular AN, which was statistically significant compared to the value in the lateral AN (control area) 0.02±0.02 (*p*<0.01), and the Rr value estimated for GLUT1/vimentin colocalization was 0.6±0.02 in the periventricular AN, which was statistically significant compared to the value in the lateral AN (control area) 0.15±0.02 (*p*<0.01). Reduced immunoreaction for MCT1 and GLUT1 was detected in tanycyte processes located in the external region of the brain (Fig. 2O–Q) and in β2-tanycytes present in the ME (Fig. 2R–U). Astrocytes detected in glial-limiting membrane (Fig. 2Q) and ME (Fig. 2U) were also positive for MCT1.

### MCT4 cellular localization in tanycytes

**Figure 3:**
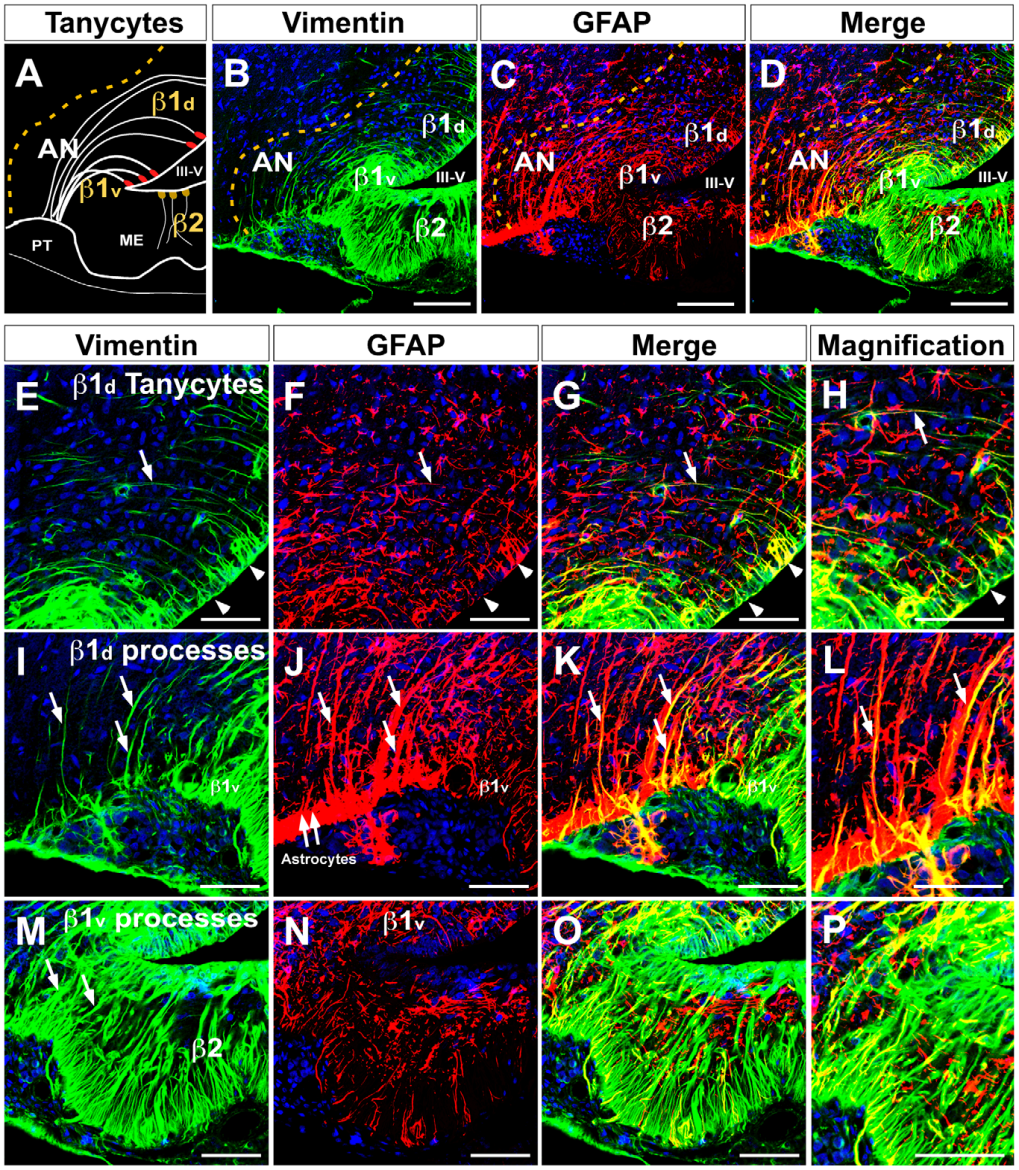
Dorsal and ventral β1-tanycytes distribution in the basal hypothalamus. **A**, Schematic representation of the hypothalamic basal area showing dorsal and ventral β1-tanycytes. **B**, Low magnification of the basal hypothalamic area using anti-vimentin antibodies (green) and the TOPRO nuclear stain (blue). **C**, Low magnification using anti-GFAP antibodies (red) and the TOPRO nuclear stain (blue). **D**, Differential distribution of vimentin and GFAP in dorsal and ventral β1-tanycytes. **E–L**, Dorsal β1-tanycytes (β1_d_) showed reduced vimentin staining. The reaction was concentrated in the ventricular area (E, head arrows), which was also positive for GFAP (F–H). The processes of these cells were strongly positive for GFAP (J–L, arrows). **M–P**, Ventral β1-tanycytes (β1_v_) showed intense immunoreaction for vimentin (M) and very low staining with anti-GFAP (N–P). AN, arcuate nucleus, III-V: third ventricle, ME: median eminence. Scale bar: B–D, 150 µm; E–G, I–K and M–O, 50 µm; H, L and P, 25 µm.

As shown in Fig. 1, MCT4 was mainly located in the lateral region of the AN. Therefore, to define the relationship between MCT4 expression and tanycyte distribution, a detailed immunohistochemical analysis using anti-vimentin and anti-GFAP antibodies in the hypothalamic basal area was undertaken. β1-tanycytes with short processes that contact the periventricular AN were primarily identified with anti-vimentin (Fig. 3A–D). However, β1-tanycytes with long processes that contact the lateral AN were mainly identified with anti-GFAP (Fig. 3A–D). Thus, two distinct groups of β1-tanycytes were identified. β1 cells with a dorsal ventricular localization (β1_d_) are positive for GFAP (processes) and have reduced immunoreaction for vimentin (apical localization) (Fig. 3E–L). Additionally, tanycytes with ventral ventricular localization (β1_v_) are mainly positive for vimentin and have reduced immunoreaction for GFAP (Fig. 3M–P). Hypothalamic astrocytes are mainly codistributed with β1_d_-tanycytes and β2-tanycytes in the ME.

**Figure 4:**
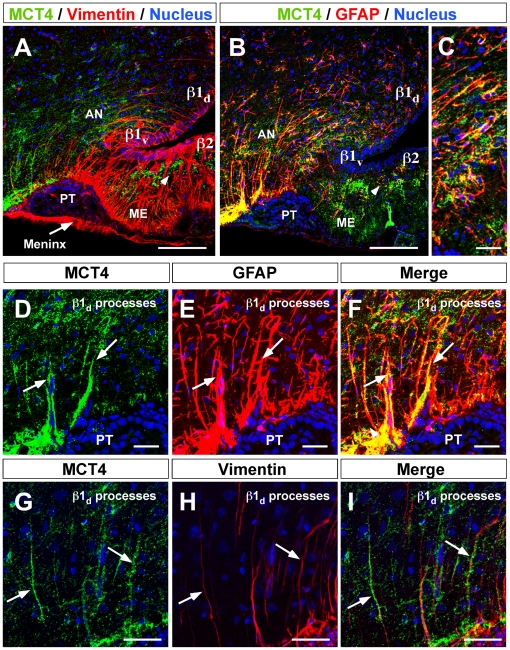
MCT4 localization in dorsal β1-tanycytes. **A**, Low magnification of hypothalamic area using anti-MCT4 antibodies (green), anti-vimentin antibodies (red), and the nuclear stain, TOPRO (blue). **B**, Low magnification using anti-MCT4 antibodies (green), anti-GFAP antibodies (red), and the TOPRO nuclear stain (blue). **C**, High magnification showing GFAP-positive processes of β1_d_-tanycytes in contact with lateral AN neurons. β1_d_-tanycytes processes were positive for MCT4 and GFAP (**D–F**). MCT4 was expressed in a number of β1-tanycyte processes, which expressed vimentin (**G–I**, arrows). AN, arcuate nucleus, III-V: third ventricle, ME: median eminence. Scale bar: A–B, 150 µm; C–I, 50 µM; J–L, 25 µm.

MCT4 (green) and vimentin or GFAP (red) colocalization was next assessed (Fig. 4A–C). MCT4 distribution was evaluated using high magnification imaging; it was detected principally in β1_d_-tanycyte processes that contact lateral AN neurons, showing strong GFAP coexpression (Fig. 4C and D–F, arrows), but low-to-absent vimentin colocalization (Fig. 4G–I, arrows). The Rr value estimated for GFAP/MCT4 colocalization was 0.5±0.01 in the lateral AN, which was statistically significant compared to the value in the periventricular AN (control area) 0.2±0.01 (*p*<0.01). Additionally, MCT4 was observed in subependymal and ME astrocytes (data not shown) and blood vessels (Fig. S1J,N).

### Functional characterization of MCT1 and MCT4 in primary cultures of tanycytes

**Figure 5:**
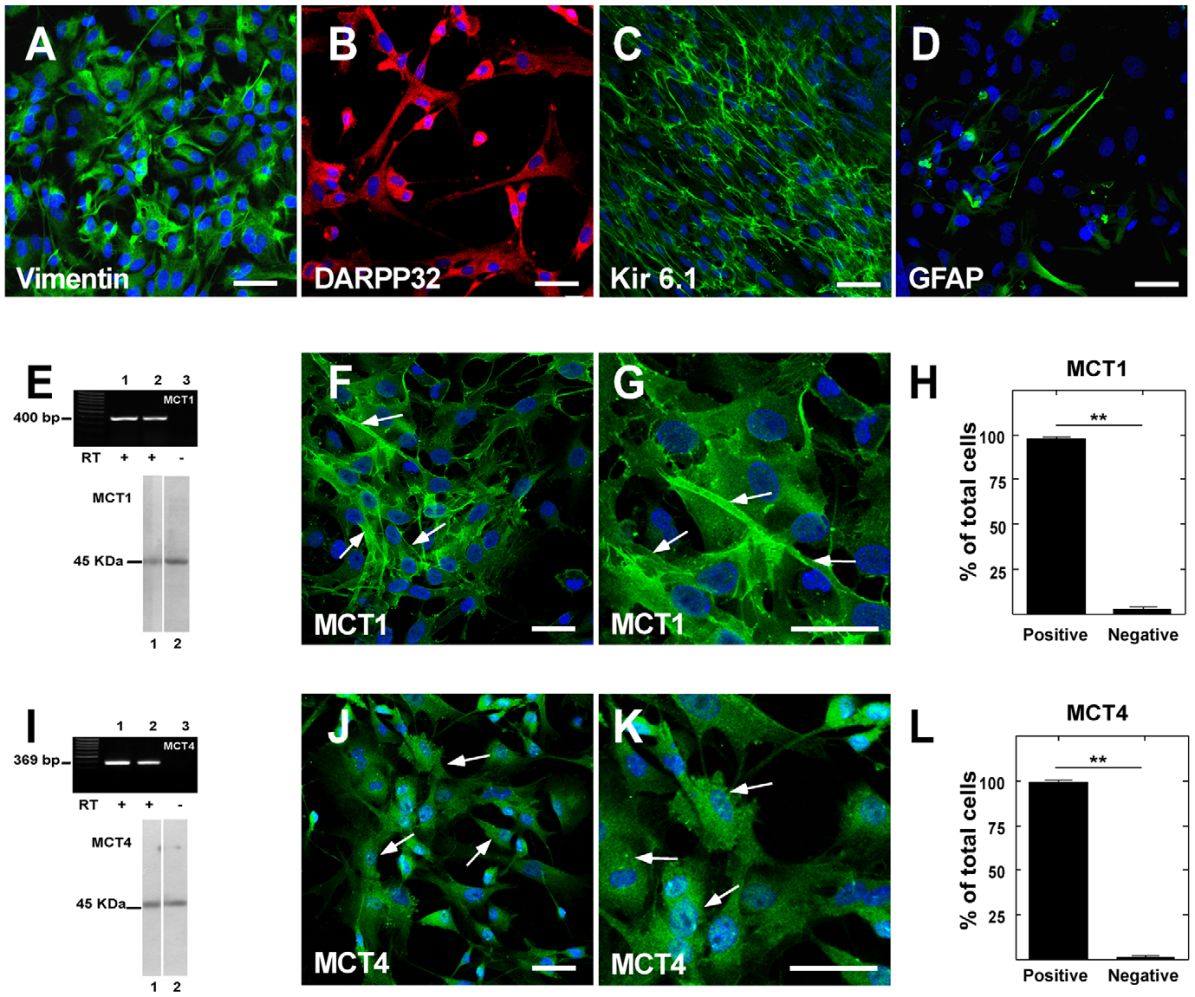
MCT1 and MCT4 expression in tanycyte cultures. **A–D**, Tanycyte cultures showed high expression of vimentin, DARPP-32, and Kir 6.1 and a small number of GFAP-positive cells. **E**, MCT1 RT-PCR (upper panel) and immunoblot (lower panel) analyses. RNA isolated from renal cortex (lane 1) and tanycyte cultures (lane 2). RT(-) of tanycyte culture (lane 3). Total protein extracted from renal cortex (lane 1) and tanycyte cultures (lane 2). **F–G**, Immunohistochemical studies using anti-MCT1 antibodies (green) and TOPRO nuclear stain (blue). **H**, Average MCT1-positive cells. **I**, MCT4 RT-PCR (upper panel) and immunoblot (lower panel) analyses. RNA isolated from skeletal muscle (lane 1) and tanycyte cultures (lane 2). Total protein extracted from skeletal muscle (lane 1) and tanycyte cultures (lane 2). **J–K**, MCT4 immunohistochemical studies using anti-MCT4 antibodies (green) and TOPRO nuclear stain (blue). **L**, Average of MCT4-positive cells. Scale bar: 25 µm.

To analyze MCT1 and MCT4 tanycyte expression, primary tanycyte cultures were obtained. Most cells exhibited a polarized morphology that consisted of a wide proximal cytoplasmic region containing the nucleus and a long basal process (data not shown). Immunohistochemical analysis revealed an intense positive reaction with anti-vimentin (Fig. 5A), anti-DARPP32 (Fig. 5B), and anti-Kir 6.1 (Fig. 5C) antibodies; however, reduced immunoreaction using anti-GFAP antibodies was observed (Fig. 5D). RT-PCR and Western blot analyses demonstrated MCT1 and MCT4 expression in tanycyte cultures (Fig. 5E and I, lane 2 respectively). Total kidney cortex or skeletal muscle RNAs or protein extracts were used as positive controls for MCT1 and MCT4 expression, respectively (Fig. 5E and I, lane 1). Immunocytochemical analysis confirmed MCT1 and MCT4 expression in tanycytes (Fig. 5F–L, arrows). An average of 92% expressed MCT1 and 94% expressed MCT4 (Fig. 5H and L).

**Figure 6:**
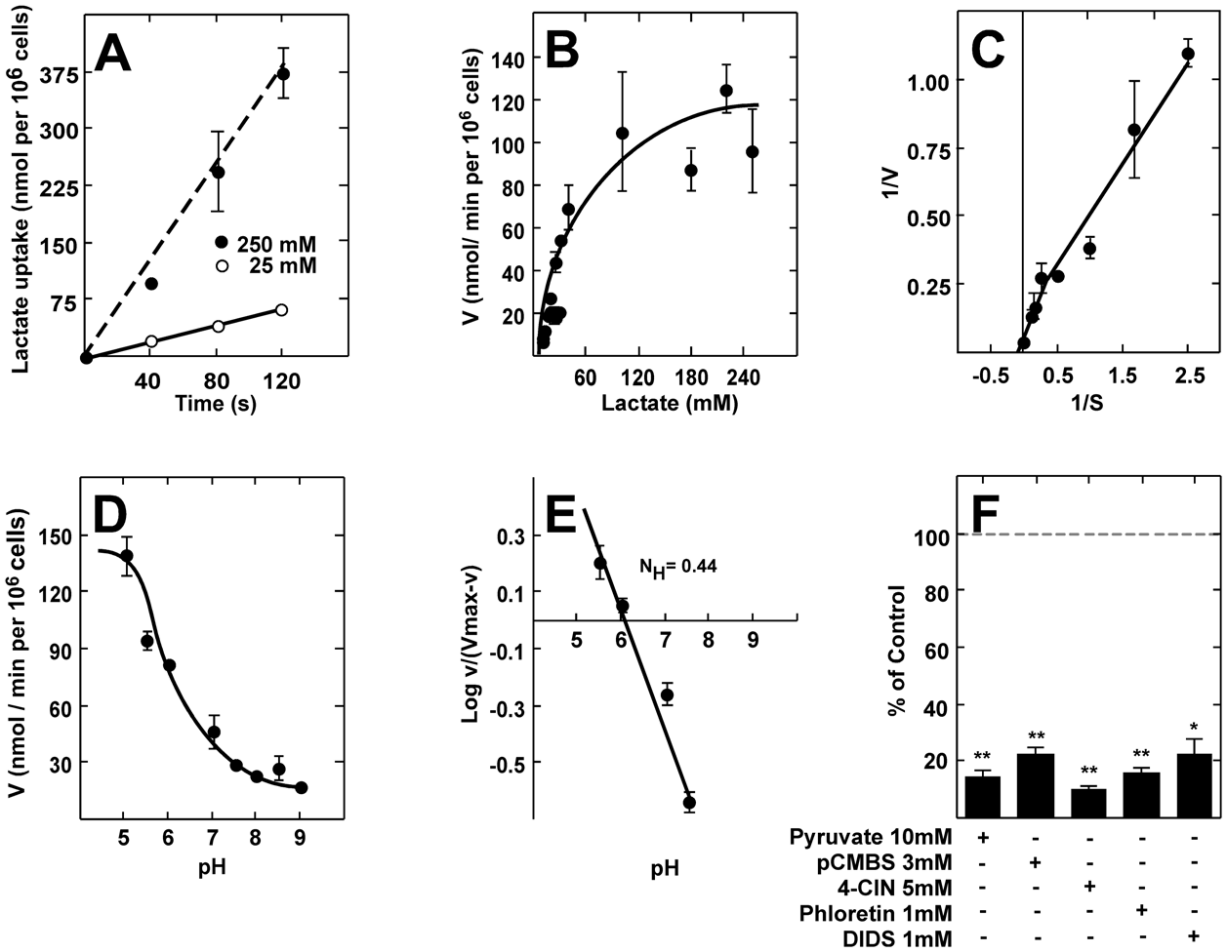
Functional characterization of cultured tanycytes. **A**, 25 mM L-lactate (open circles) and 250 mM L-lactate (closed circles) transport at 4°C and pH 7.0 over time. **B**, Kinetic parameters of L-lactate transport in tanycyte cultures at 1 min, 4°C, and pH 7.0. **C**, Double-reciprocal plot of MCT1: (Km, 6 mM; Vmax, 13 nmol×10^6^ cells/min) and MCT4: (Km, 48 mM; Vmax, 100 nmol×10^6^ cells/min). **D**, Dependence of lactate uptake on pH (0.1 mM L-lactate, 4°C). **E**, Hill plot to analyze the dependence of lactate uptake on pH. **F**, Analysis of lactate transport in the presence of various inhibitors co-incubated for 1 min (0.1 mM lactate, 4°C, pH 7.0). Results represent the mean ± SD of three independent experiments. **p<0.001, one tailed t-test.

The uptake of radioactive lactate was used to analyze the functional activity of MCTs in tanycyte cultures. To further reduce the effects of metabolism and transamination of pyruvate to alanine, short incubation times and low temperatures were used in all experiments. The uptake of 25 and 250 mM lactate was almost linearly correlated with time up to 120 s at 4°C (Fig. 6A). Using these optimized conditions, the basic transport parameters for lactate uptake in primary cultures of rat tanycytes were determined. Dose-response studies using 1-min assays revealed that lactate transport was saturated at concentrations above 150 mM (Fig. 6B). Data analysis suggested the presence of two kinetic components for lactate transport. When the data were transformed and plotted according to Lineweaver-Burk, this observation was confirmed (Fig. 6C). Transformation showed apparent Km values of 6 mM and 48 mM, and Vmax values of 13 nmol/(min×10^6^ cells) and 100 nmol/(min×10^6^ cells), corresponding to MCT1 and MCT4, respectively. The uptake of lactate in cultured tanycytes increased with increasing H^+^ concentration. At a substrate concentration of 0.1 mM, lactate uptake at pH 6.0 was more than three times faster than that observed at pH 8.0 (Fig. 6D). The proton effect was found to be of a non-cooperative nature, which was corroborated by a Hill plot that yielded a straight line with a slope (Hill coefficient) of 0.44 (Fig. 6E). To determine if other monocarboxylates might also serve as substrates for the lactate transporter in tanycytes, competition experiments were performed. At an extracellular concentration of 0.1 mM lactate (4°C, pH 7.0), transport was strongly inhibited by 10 mM pyruvate. Incubation with MCT inhibitors, pCMBS and 4-CIN, decreased lactate uptake up to 80% and 90% respectively; floretin and DIDS also potently inhibited lactate uptake (Fig. 6F).

**Figure 7:**
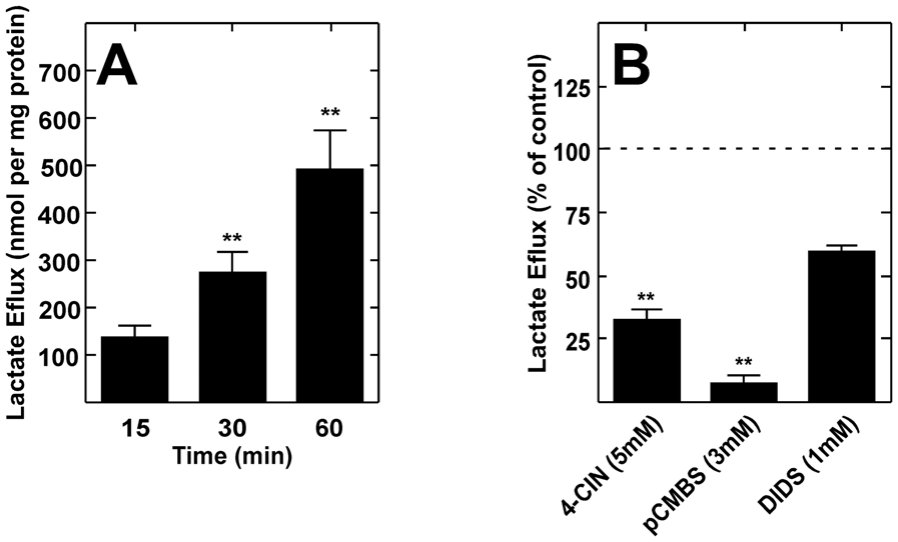
Cultured tanycytes release lactate. **A**, Lactate efflux at 5 mM glucose. Lactate efflux increased throughout the incubation time. **B**, Analysis of lactate efflux in the presence of several inhibitors pre-incubated for 15 min at 37°C. Lactate efflux decreased significantly with 4-CIN and pCMBS. **p<0.001, one tailed t-test. Results represent the average ± SD of three independent experiments.

Because tanycytes may uptake lactate using MCT1 and MCT4, lactate efflux was also examined. Specifically, the cultured tanycytes were incubated with 5 mM glucose for 15, 30, and 60 min. Lactate release was evaluated by HPLC of cell culture supernatant. After incubation with glucose for 15 min, 150 nmol lactate/mg protein was detected; however, this value was two-fold increased after 30 and 60 min (Fig. 7A). These results demonstrate that glucose induces lactate efflux in cultured tanycytes. Lactate efflux generated by 5 mM glucose (30 min) was inhibited 50% by 5 mM 4-CIN and 80% by 3 mM pCMBS; DIDS did not significantly inhibit lactate efflux (Fig. 7B).

## Discussion

Hypothalamic glial cells known as α and β-tanycytes are specialized ependymal cells that bridge the CSF and the neuroendocrine neurons localized in the AN. Tanycytes express GLUT2 and GK, two proteins known to participate in glucosensing mechanisms [29], [30]. Injection of alloxan, a GK inhibitor, into the III-V impairs feeding behavior and blood glucose concentration in treated rats [33]. These impaired responses are thought to be associated with the destruction of III-V tanycytes, neuronal swelling and decreased AN neuropeptide Y mRNA. However, once the alloxan-induced tanycyte destruction is reversed, feeding behavior was restored [33]. In this study, we demonstrate that tanycytes express MCT1 and MCT4, and both isoforms are involved in the influx and efflux of lactate. Studies performed in ventromedial hypothalamic slices have shown that glucose-responsive neurons also respond to changes in lactate concentration [4], [6]. Intracerebro-ventricular (ICV) infusion of either glucose or lactate is sufficient to decrease blood glucose level through the inhibition of liver glucose production [34]. Interestingly, the coinfusion of glucose or lactate with oxamate, an inhibitor of lactic dehydrogenase, in the III-V abolished the effects of both ICV-glucose and ICV-lactate on blood glucose levels, demonstrating that the conversion of glucose to lactate and subsequent generation of pyruvate are required for changes induced by ICV-glucose [34]. Moreover, the selective distribution of MCTs between neurons and astrocytes suggests that lactate plays a key role in brain energy metabolism. In particular, MCT1 and MCT4 would be essential to ensure lactate release. However, to date, no information is available concerning the expression of MCTs in tanycytes, the main glial cell present in the AN [28], [29], [30].

Our detailed immunohistochemical and functional analyses of MCT isoforms, both *in situ* and *in vitro*, indicated that MCT1 is expressed in the β1 tanycytes of adult rats. Previous localization studies of MCTs in the hypothalamus have only reported their expression in ciliated ependymal cells [18], [20], [35] and astrocytes [12]. The data reported here represent the first evidence that hypothalamic tanycytes highly express these transporters. MCT1 expression was mainly observed in the blood-brain barrier endothelial cells, astrocytes from several regions, and in the brain marginal zone, which is in agreement with previous findings [12], [17], [18], [19], [20], [35], [36]. Moreover, MCT4 expression was detected in astrocytes from hippocampus, *corpus callosum* and cortex [12], [25].

Q-RT-PCR analysis revealed that MCT1 is more highly expressed than the MCT4 isoform in the hypothalamic tissue, which was confirmed by immunohistochemical analyses. Specifically, MCT1 was localized to the periventricular area whereas MCT4 expression was observed in the lateral area of the III-V. Furthermore, MCT1 expression was localized preferentially in cell bodies and processes of β1_v_-tanycytes, without any cellular polarization. These tanycytes may be in contact with neuropeptide Y-expressing neurons (periventricular area) and affect the activity of GI neurons [37], [38]; however, further studies are needed to demonstrate this hypothesis.

MCT4 expression was demonstrated in hypothalamic β1_d_-tanycytes in the present study. Specifically, MCT4 expression was restricted to GFAP-positive processes contacting lateral AN neurons. The MCT4 distribution suggests a preferential sorting of this transporter to the β1_d_-tanycytes processes that contact GE neurons in the lateral AN (POMC-positive neurons in mouse brain) [39], [40]. It is possible that lactate released by β1_d_-tanycytes impacts POMC neurons, stimulating satiety. Accordingly, it has been recently demonstrated that central administration of lactate lowers food intake and body weight in rats [41]. To demonstrate the functional expression of the lactate transporters, primary hypothalamic glial cell cultures were obtained. The cell cultures presented a highly elongated and polarized form similar to tanycytes observed *in vivo*. These cells were immuno-positive for three tanycyte markers, vimentin, DARPP32, and Kir 6.1 [29], [42], and to a minor degree for GFAP. Immunohistochemical analysis of MCT expression showed the presence of MCT1 and MCT4 in cultured cells. These findings are analogous to what is observed *in vivo*. RT-PCR and Western blot analyses confirmed the expression of MCT1 and MCT4 in cultured tanycytes, and molecular weight analyses revealed that tanycytes expressed a 43-kDa MCT isoform. Overall, the immunolocalization and immunoblotting data strongly support the concept that the cells isolated from the hypothalamic area and grown *in vitro* corresponded to primary cell cultures enriched in differentiated hypothalamic tanycytes.

Transport and competition assays revealed that tanycytes express two functionally active transporters directly involved in lactate uptake. The higher affinity transport component had the expected properties of MCT1, with a transport Km of 6 mM [16]. In contrast, kinetic analysis of the lower affinity transporter revealed an apparent Km of approximately 34 mM, which fits the description of MCT4 using radiolabeling techniques [15]. The uptake of lactate in cultured tanycytes increased with increasing H^+^ concentration; the proton effect was of a non-cooperative nature as demonstrated with a Hill coefficient of 0.44, which has been reported to be the value for MCT1 and MCT4 [15], [16]. Lactate transport was strongly inhibited by 10 mM pyruvate as both isoforms transport pyruvate [15], [43]. Additionally, 5 mM 4-CIN decreased lactate uptake by 80% as was expected since 4-CIN displays an IC_50_ of approximately 350 µM for lactate uptake in both isoforms [15], [43]. Floretin also strongly inhibited lactate uptake, which is similar to that observed in COS cells that express both MCT isoforms [44]. In addition, a smaller but significant inhibitory effect on lactate uptake was observed with 1 mM DIDS, which was similar to that observed in MCT4-expressing oocytes [15]. Specifically, a maximal inhibition of 60% was obtained even after the addition of millimolar concentrations of this inhibitor, which completely inhibits MCT1-mediated lactate uptake [15]. Therefore, kinetic data and sensitivity to inhibitors confirmed that these transporters corresponded to MCT1 and MCT4, coinciding with the immunohistochemical and RT-PCR data.

In order to support the metabolic interaction between tanycytes and AN neurons, lactate efflux analysis from isolated tanycytes was performed. Physiological concentration of glucose induced lactate efflux in cultured tanycytes, which was similar to the lactate efflux reported in astrocytes exposed to glutamate [45]. The sensitivity to pCMBS is a distinct characteristic of specific members within the MCT family; this molecule inhibits MCT1 and MCT4, but not MCT2 [43]. More than 90% inhibition for influx and efflux was observed, confirming that MCT1 and MCT4 are involved in both mechanisms. DIDS did not significantly alter lactate efflux, confirming MCT4 participation since MCT1 is completely inhibited by DIDS [15]. Taken together, these findings demonstrated the capacity of tanycyte cultures to release lactate through MCT.

Participation of lactate in glucosensing and feeding behaviour is supported by several studies [9], [34], [46]. Central inhibition of lactate dehydrogenase by administration of oxamate abolishes the effects of ICV-glucose on blood glucose-lowering. Thus, blocking lactate metabolism in the hypothalamus results in a 40% reduction in the inhibitory action of glucose on circulating glucose levels [34], and ICV injection of lactate reduces food intake, resulting in body weight loss in eight-week-old rats [41]. Furthermore, local lactate perfusion of the VMH suppresses the hypoglycaemic counter-regulatory response, with a strong diminution in glucagon and epinephrine release [46]. Ainscow et al. [9] performed dynamic bioluminescence imaging to record [ATP]c in real-time during glucose or lactate challenge of neurons and glial cells. Using adenovirus-based vectors to express luciferase with high efficiency in neurons and associated glia, [ATP]c changes in hypothalamic neurons in response to elevations in glucose concentration are below the level of detection for this assay (≈2%); however, neurons respond to lactate with a significant increase in [ATP]c [9]. These findings provide evidence for both a direct intracellular mechanism of K_ATP_ channel regulation, which does not involve increases in [ATP]c [8], as well as a separate intercellular signalling mechanism mediated by lactate released from neighbouring glial cells.

The specific localization of tanycytes in direct contact with CSF and GLUT2/GK expression strongly support the idea that these cells have a high capacity to uptake glucose. Therefore, tanycytes could uptake glucose, metabolize it to lactate through the glycolytic pathway, and subsequently release it through MCT1 and/or MCT4, thus providing neighbouring neurons with information regarding glucose levels and regulating glucosensing activities.

## Materials and Methods

### Ethics statement

All animals were handled in strict accordance with the Animal Welfare Assurance (permit number 2010101A) and all animal work was approved by the appropriate Ethics and Animal Care and Use Committee of the University of Concepcion, Chile. Male adult Sprague-Dawley rats were used for the experiments. Animals were kept in a 12-h light/dark cycle with food and water *ad libitum*.

### Immunocytochemistry

Rats were fixed in Bouin solution (750 mL of saturated picric acid, 250 mL of formaldehyde 37%, and 50 mL of glacial acetic acid) or 4% paraformaldehyde (PFA) using vascular perfusion, and the samples were dissected and post-fixed by immersion (12 h). After post-fixation, the samples were dehydrated in graded alcohol solutions and embedded in paraffin. Sections of the kidney and skeletal muscle (7 µm) were obtained and mounted on poly-L-lysine-coated glass slides. Alternatively, thick frontal sections of the hypothalamus ( µm, fixed in PFA) were cut with a cryostat, and subsequently processed free-floating. Before immunostaining, the sections undergoing peroxidase immunohistochemistry were treated with 3% hydrogen peroxide in absolute methanol to inactivate endogenous peroxidase activity.

For immunohistochemical analyses, the following antibodies and dilutions were used: rabbit anti-GLUT1 (1∶100, Alpha Diagnostic International, INC., San Antonio, TX, USA), rabbit anti-glial fibrillary acidic protein (GFAP; 1∶200, DAKO, Campintene, CA, USA), mouse anti-vimentin (1∶200, DAKO), chicken anti-MCT1 (1∶100, Millipore, Temecula, CA, USA), rabbit anti-MCT4 (1∶20, Millipore). The antibodies were diluted in a Tris-HCl buffer (pH 7.8) containing 8.4 mM sodium phosphate, 3.5 mM potassium phosphate, 120 mM sodium chloride, and 1% bovine serum albumin. Sections were incubated with the antibodies overnight at room temperature in a humid chamber. After extensive washing, the sections were incubated for 2 h at room temperature with peroxidase-labeled anti-chicken IgY (1∶500; Jackson ImmunoResearch Laboratories, INC., Pennsylvania, USA). The peroxidase activity was developed using a DAB substrate kit (ImmunoPure; PIERCE Biotechnology, Rockford, IL, USA). For immunofluorescence and colocalization analyses, the tissues were incubated with the primary antibodies overnight and subsequently with Cy2-, Cy3- or Cy5-labeled secondary antibodies (1∶200; Jackson ImmunoResearch Laboratories). These samples were counter-stained with the DNA stain, TOPRO-3 (1∶1000; Invitrogen, Rockville, MD, USA). The slides were analyzed using confocal laser microscopy (D-Eclipse C1 Nikon, Tokyo, Japan).

### Image analysis

To analyze the colocalization levels for MCT1/vimentin, MCT1/GLUT1, vimentin/GLUT1, and MCT4/GFAP immunostaining within various regions of interest in the periventricular AN (β1_v_-tanycytes) or hypothalamic lateral region (β1_d_-tanycytes), Pearson's coefficient (Rr) values were calculated using the NIS-Elements software (Nikon, Nikon Instruments INC). This coefficient measures the overlapping level between the pixels of two fluorescent channels, ranging from −1 to +1 (0–100% colocalization) [47]. The statistical analysis was performed compared to interest region with a control region using Student's t-test.

### Reverse transcription-polymerase chain reaction

The brain of each rat was removed, and the hypothalamic area was isolated and further dissected to obtain a region close to the ependymal layer. Total RNA from hypothalamus, control tissues, or cell cultures were isolated using Trizol (Invitrogen). For RT-PCR, 2 µg RNA was incubated in a 20 µL reaction volume containing 5X buffer for M-MulV reverse transcriptase, 20 U RNAse inhibitor, 1 mM dNTPs, 2.5 µM oligo(dt)18 primer, and 10 U revertAidTM H minus M-MuLV reverse transcriptase (Fermentas International INC., Burlington, Ontario, Canada) for 5 min at 37°C followed by 60 min at 42°C and 10 min at 70°C. Parallel reactions were performed in the absence of reverse transcriptase to control for the presence of contaminant DNA. For amplification, 1 µL cDNA aliquot in a total volume of 12.5 µL containing 10X PCR buffer without MgCl_2_, 10 µM dNTPs, 25 µM MgCl_2_, 0.3125 U Taq DNA pol (Fermentas International), and 10 µM of each primer was incubated at 95°C for 5 min followed by 35 cycles of 30 s at 95°C, 30 s at 55°C, and 30 s at 72°C and a final extension of 7 min at 72°C. PCR products were separated by 1.2% agarose gel electrophoresis and visualized by staining with ethidium bromide. The following sets of primers were used: MCT1, sense 5-′GGG AAG GTG GAA AAA CTC AA-3′ and antisense 5′-ACA CTC CAT TCG CAA CAA CA-3′ (expected product of 400 bp); MCT4, sense 5′-TGC GGC CCT ACT CTG TCT AC-3′and antisense 5′-TCT TCC GAT GCA GAA GAA G-3′ (expected product of 369 bp); and β-actin, sense 5′-GCT GCT CGT CGA CAA CGG CTC-3′ and antisense 5′-CAA ACA TGA TCT GGG TCA TCT TCT C-3′ (expected product of 353 bp).

### Real time PCR

Total RNA samples were treated with DNase I before reverse transcription processing to remove genomic DNA contamination. A total of 2 µg RNA from each sample was reverse transcribed into cDNA using the protocol described above. Q-RT-PCR reactions were prepared with a Brilliant II SYBR Green QPCR Master Mix kit (Agilent Technologies, Inc., Santa Clara, CA, USA) in a final volume of 12.5 µL containing 1 µL cDNA, 500 nM primers, and 30 nM ROX dye. PCR reactions were carried out in an Mx3000P QPCR System (Agilent Technologies). The following sets of primers were used: cyclophilin (the housekeeping gene), sense 5′-ATA ATG GCA CTG GTG GCA AGT C-3′and antisense 5′-ATT CCT GGA CCC AAA ACG CTC C-3′ (expected product of 239 bp); MCT1, sense 5′-TGG AAT GTT GTC CTG TCC TCC TGG-3′ and antisense 5′-TCC TCC GCT TTC TGT TCT TTG GC-3′ (expected product of 178 bp); and MCT4, sense 5′-TTC TCC AGT GCC ATT GGT CTC GTG-3′ and antisense 5′-CCC GCC AGG ATG AAC ACA TAC TTG-3′ (expected product of 122 bp). The thermal cycling conditions consisted of a 10 min denaturation period at 95°C, followed by 40 cycles of denaturation for 30 s at 95°C, annealing for 30 s at 55°C, and extension for 1 min at 72°C. The relative expression of MCT to cyclophilin mRNA was calculated on the basis of the PCR efficiency. The statistical analysis was performed using GraphPad Prism 4.0 Software (GraphPad Software Inc., San Diego CA). MCT expression was compared using the Student's t-test.

### Immunoblotting

Total protein extracts were obtained from rat hypothalamus, kidney, and skeletal muscle as well as primary cultures of tanycytes. Tissues and cells were homogenized in buffer A (0.3 mM sucrose, 3 mM DTT, 1 mM EDTA, 100 µg/mL PMSF, 2 µg/mL pepstatin A, 2 µg/mL leucopeptin, and 2 µg/mL aprotinin), sonicated three times on ice at 300 W (Sonics & Material INC, VCF1, Connecticut, USA) for 10 s, and separated by centrifugation at 4000×g for 10 min. Proteins were resolved by SDS-PAGE (50 µg/lane) in a 5–15% (w/v) polyacrylamide gel, transferred to PVDF membranes (0.45 µm pore, Amersham Pharmacia Biotech., Piscataway, NJ, USA), and probed for 2 h at 4°C with chicken anti-MCT1 (1∶1000) or rabbit anti-MCT4 (1∶500) antibodies. After extensive washing, the PVDF membranes were incubated for 1 h at 4°C with peroxidase-labeled anti-chicken IgY (1∶1000; Jackson Immuno Research) or peroxidase-labeled anti-rabbit IgG (1∶5000; Jackson Immuno Research). The reaction was developed using the enhanced chemiluminescence (ECL) Western blot analysis system (Amersham Biosciences, Pittsburgh, PA, USA). Negative controls consisted of incubating the membrane with a pre-absorbed antibody (anti-MCT1 1∶100 or MCT4 1∶500 with 100 µg/mL inductor peptide incubated at 4°C overnight).

### Cell culture

Hypothalamic glial cell cultures from 1-day postnatal brain were isolated following the method described previously [29], [48]. Briefly, the brain and specifically the hypothalamic area was removed and further dissected to obtain a region close to the ependymal layer. The dissection was carried out with the samples submerged in dissection buffer containing 10 mM HEPES (pH 7.4, 340 mOsm/L). Samples were incubated with 0.25% trypsin-0.2% EDTA (w/v) for 20 min at 37°C. Trypsinized tissue was transferred to planting medium containing MEM, (Invitrogen) with 10% (v/v) fetal bovine serum (FBS) (Thermo Fisher Scientific Inc., Waltham, MA, USA), and 2 mg/mL DNAse I (Sigma-Aldrich, St. Louis, MO, USA). Cells were seeded at 1.2×10^5^ cells/cm^2^ in culture dishes treated with 0.2 mg/mL poly-L-lysine (Sigma-Aldrich). After 2 h, the culture medium was changed to MEM supplemented with 10% FBS, 2 mM L-glutamine, 100 U/mL penicillin, 100 µg/mL streptomycin, and 2.5 µg/mL fungizone (Thermo Fisher Scientific Inc). Cells were cultured in the same dish for 3 weeks, and the medium was changed every 2 days. The dishes with the highest density of confluent epithelial cells were expanded and used for immunocytochemistry, lactate uptake, and efflux experiments.

For immunocytochemistry, cells were grown on poly-L-lysine-coated glass cover slides in 24-well plates, fixed with 4% paraformaldehyde in PBS for 30 min, washed with Tris-HCl buffer (pH 7.8), and incubated in the same buffer containing 1% bovine serum albumin (BSA) and 0.2% Triton X-100 for 5 min at room temperature. Samples were then incubated with the following primary antibodies overnight at room temperature: chicken anti-MCT1 (1∶100), rabbit anti-MCT4 (1∶20), goat anti- dopamine- and cyclic AMP-regulated phosphoprotein-32 (DARPP-32; 1∶100, Santa Cruz Biotechnology, Santa Cruz, CA, USA), goat anti-Kir6.1 (1∶50, Santa Cruz Biotechnology), rabbit anti-GFAP (1∶200), mouse anti-vimentin (1∶200). Cells were then incubated with Cy2- or Cy3-labeled secondary antibodies and counter-stained with the DNA stain, TOPRO-3 (1∶1000, Invitrogen). The slides were analyzed using confocal laser microscopy (D-Eclipse C1 Nikon, Tokyo, Japan).

### Uptake analysis

For lactate uptake assays, cells were seeded in 12-well plates (2×10^5^ cells/well) and grown for 15 days to confluence. Cultures were carefully selected under the microscope to ensure that only plates showing uniformly growing cells were used. For each experiment, six wells were used to quantify the number present in each well. No significant variations in the number of cells were observed after incubation with incubation buffer [48], [49]. Cells were washed and placed in incubation buffer (15 mM HEPES [pH 7.0], 135 mM NaCl, 5 mM KCl, 1.8 mM CaCl_2_, 0.8 mM MgCl_2_, 320 mOsm) for 10 min at room temperature. Uptake assays were performed in 0.2 mL of incubation buffer at 4°C with various L-lactate (Sigma-Aldrich) concentrations (0.1 to 250 mM) and 1-4 µCi of L-[14C(U)]lactic acid sodium salt (>100 mCi [3.70GBq]/mmol; Perkinelmer-NEN, Boston, MA, USA). Uptake was stopped by washing the cells with ice-cold stop buffer (incubation buffer plus 1 mM HgCl_2_). Cells were lysed in 0.5 mL of lysis buffer (10 mM Tris-HCl [pH 8.0], 0.2% SDS), and the incorporated radioactivity was quantified by liquid scintillation counting. When appropriate, lactate uptake was assessed at several pH levels, and inhibitors were used. In L-lactate inhibition experiments, cells were pre-incubated with alpha-cyano-4-hydroxycinnamate (4-CIN, Sigma-Aldrich), p-chloromercuribenzene sulfonate (pCMBS, Sigma-Aldrich), floretin (Sigma-Aldrich), or di-isothiocyanostilbene disulfonate (DIDS, Sigma-Aldrich) for 15 min at 37°C (incubation buffer). For competitive analyses, cells were co-incubated with L-pyruvate at 4°C. All inhibition experiments were carried out under or at initial velocity conditions to discriminate between L-lactate transport and metabolism. In all assays, the incubation buffer was adjusted to 320 mOsm; when osmolarity could not be adjusted at this value; controls with the same osmolarity were used. In inhibition experiments, statistical comparison between two groups of data was carried out using Student's t-test.

### Efflux analysis

For lactate release assays, cells were seeded in 12-well plates (2×10^5^ cells/well) and grown for 15 days to confluence in 2 mM glucose. Cells were washed with 0.1 M PBS (10.6 mM Na_2_HPO_4_, 3.2 mM KH_2_PO_4_, 123.5 mM NaCl, pH 7.4 and 320 mOsm) and incubated for several times in efflux buffer (44 mM sucrose, 10 mM HEPES [pH 7.4], 135 mM NaCl, 5 mM KCl, 0.15 mM Na_2_HPO_4_, 0.2 mM KH_2_PO_4_ and 5 mM glucose). Supernatant was removed and frozen in liquid nitrogen until quantification. Cells were lysed in buffer A and sonicated three times on ice at 300 W for 10, and total protein concentration was determined using the Bradford assay (Bio-Rad Laboratories, Hercules, CA, USA). 4-CIN, pCMBS, and DIDS inhibitors were pre-incubated with the cells for 15 min at 37°C. Data represent means ± SD of three experiments performed in duplicate. The statistical analysis was performed using Student's t-test.

Supernatant was assayed for lactate using a high performance liquid chromatography (HPLC) system from Merck Hitachi (Merck, Darmstadt, Germany), consisting in an L-6200 pump and a Hitachi L-4200 UV-VIS (225 nm) detector. Samples were separated by chromatography on a Aminex HPX-87H column (Bio-Rad Laboratories, Hercules, CA, USA) of 300×7.8 mm I.D., connected with a standard cartridge holder 30×4.6 mm I.D. to protect the analytical column. The mobile phase consisted of an isocratic solution of 20 mM H_2_SO_4_. The solvent flow rate was 0.5 mL/min, and the backpressure was lower than 1000 psi. The L-lactate peak was identified by comparison of its retention time with that of a reference standard, and its concentration was quantified using the area under the peak (Merck Hitachi D-2500 chromato integrator).

## Supporting Information

Figure S1 **MCT1 and MCT4 immunolocalization.** **A**, MCT1 localization in kidney slices. A positive reaction was observed in the cortical S1 proximal convoluted tubule (PCT, arrows). **B–G**, MCT1 localization in the nervous system. MCT1 was detected in capillaries of the hippocampus (B, arrowhead), ependymal cells (C, arrow); satellite cells (D, arrows) and astrocytes (E-G, arrows). Additionally, MCT1 colocalized with GLUT1 in some endothelial cells of the cerebral cortex (F, arrowhead). **H**, MCT4 localization in skeletal muscle fibers. **I–N**, MCT4 localization in the nervous system. MCT4 was detected in astrocytes of the entorhinal cortex (I, arrows) and in blood vessels (J, arrow). To confirm the presence of MCT4 in astrocytes, double-labeling with GFAP was performed. MCT4-GFAP colocalization was seen in astrocytes end-feet of the *corpus callosum* (K, arrow), hypothalamus (L, arrows), hippocampus (M, arrows), and cerebral cortex (N, arrows). PCT, proximal convoluted tubule; G, glomeruli; S1, Segment 1. Scale bar 50 µm.
(TIF)
