# Supplementary figures and images for: MCT Expression and Lactate Influx/Efflux in Tanycytes Involved in Glia-Neuron Metabolic Interaction
Source: PLoS One. 2011 Jan 28;6(1):e16411. doi: 10.1371/journal.pone.0016411 (PMC3030577; doi:10.1371/journal.pone.0016411)

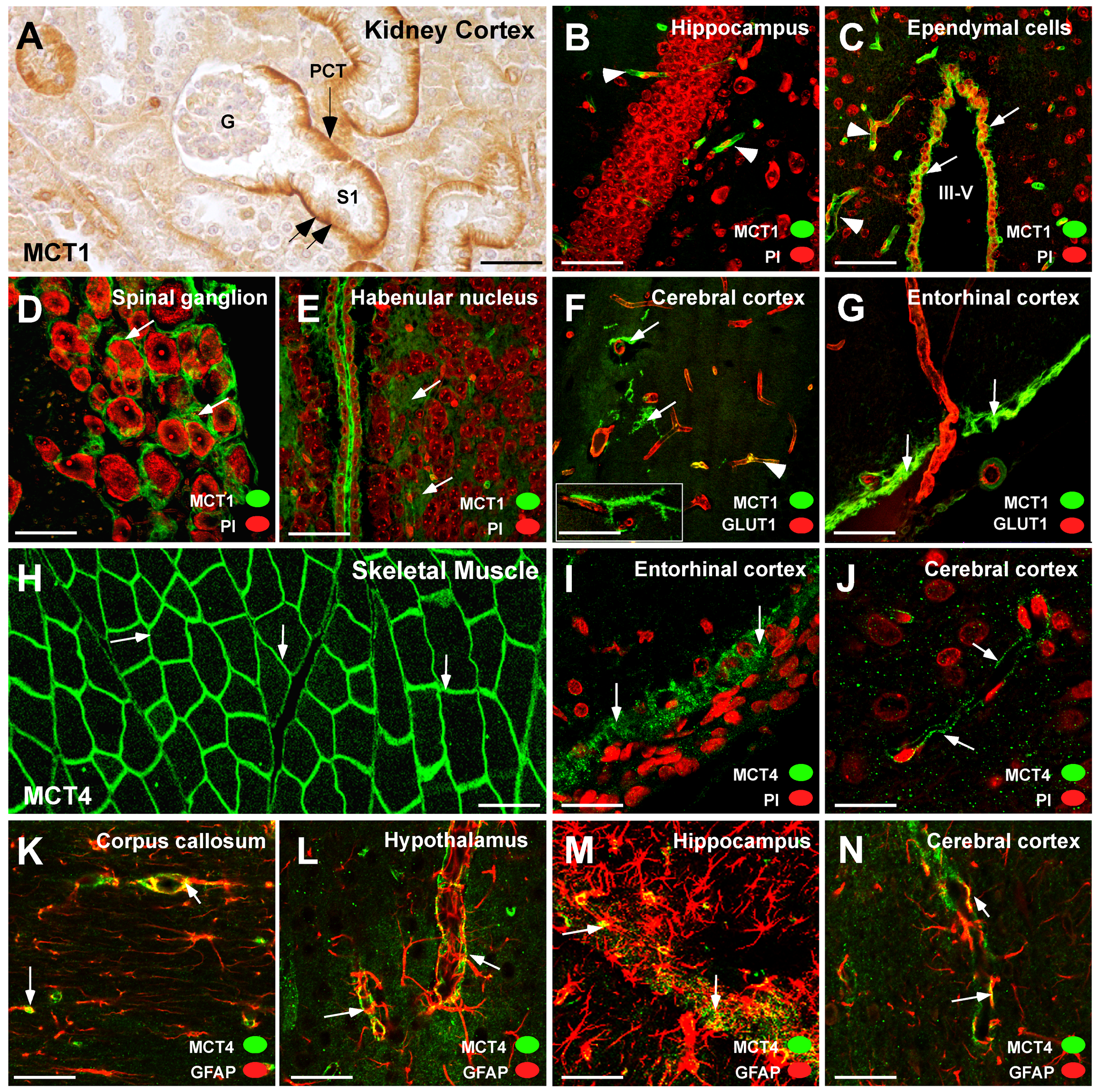

Supplement: Figure S1 — MCT1 and MCT4 immunolocalization. A, MCT1 localization in kidney slices. A positive reaction was observed in the cortical S1 proximal convoluted tubule (PCT, arrows). B–G, MCT1 localization in the nervous system. MCT1 was detected in capillaries of the hippocampus (B, arrowhead), ependymal cells (C, arrow); satellite cells (D, arrows) and astrocytes (E-G, arrows). Additionally, MCT1 colocalized with GLUT1 in some endothelial cells of the cerebral cortex (F, arrowhead). H, MCT4 localization in skeletal muscle fibers. I–N, MCT4 localization in the nervous system. MCT4 was detected in astrocytes of the entorhinal cortex (I, arrows) and in blood vessels (J, arrow). To confirm the presence of MCT4 in astrocytes, double-labeling with GFAP was performed. MCT4-GFAP colocalization was seen in astrocytes end-feet of the corpus callosum (K, arrow), hypothalamus (L, arrows), hippocampus (M, arrows), and cerebral cortex (N, arrows). PCT, proximal convoluted tubule; G, glomeruli; S1, Segment 1. Scale bar 50 µm. (TIF) [file pone.0016411.s001.tif]
